# Safety and Efficacy of Concomitant Intensive Systolic Blood Pressure Control with Anti‐amyloid Monoclonal Antibodies in Reducing Amyloid Related Imaging Abnormalities

**DOI:** 10.1002/alz70861_108913

**Published:** 2025-12-23

**Authors:** Jeremy J. Pruzin

**Affiliations:** ^1^ Banner Alzheimer's Institute, Phoenix, AZ USA

## Abstract

**Background:**

The amyloid‐targeting treatments (ATT) donanemab and lecanemab reduce amyloid plaques and slow clinical decline in early Alzheimer’s disease (AD) but are frequently complicated by amyloid‐related imaging abnormalities (ARIA), which include ARIA‐E (edema) and ARIA‐H (microhemorrhages and superficial siderosis). ARIA, especially ARIA‐E, is the most significant challenge in optimizing the safety and tolerability of ATT, especially in persons who are at high risk for AD, *APOE ε4* carriers, with homozygotes having about a 40% risk of ARIA‐E. Known modifiable risk factors for ARIA are related to blood pressure (BP). *Post‐hoc* analyses from donanemab trials showed that baseline mean arterial pressure (MAP) >107 mm Hg, roughly equivalent to a BP of ∼140/90, was associated with a 70% increased risk of ARIA‐E, while baseline antihypertensive medication use reduced this risk by 40%. 63% of Medicare beneficiaries have hypertension (SBP>130 mm Hg), and the SPRINT trial demonstrated that intensive systolic BP (SBP) control (<120 mm Hg, below current recommendation of <130 mm Hg) is safe and well‐tolerated in older adults. It remains unclear whether combining ATT with intensive SBP control could reduce ARIA‐E incidence.

**Method:**

We propose a randomized, open‐label trial of 800 persons meeting ATT eligibility criteria with screening SBP ≥130 mm Hg. Participants will be randomized 1:1 to receive ATT with usual hypertension care or ATT with intensive SBP control (<120 mm Hg). Serial brain MRI scans (baseline, 4, 8, 12, 24, 36 and 46 weeks) will be obtained to assess ARIA outcomes, adjudicated by a blinded neuroradiology panel.

**Result:**

The primary outcome is ARIA‐E incidence. Secondary outcomes include severity of ARIA‐E, ARIA‐H, symptomatic ARIA, time to resolution of ARIA, and ATT discontinuation due to ARIA. Safety will be closely monitored and assessed. Exploratory outcomes include cognition, function, and plasma biomarkers (ptau217, NfL, Aβ 42/40, GFAP, PlGF).

**Conclusion:**

This trial will determine whether intensive SBP control reduces ARIA risk in patients receiving ATT for early AD.

